# RHOXF2 gene, a new candidate gene for spermatogenesis failure

**DOI:** 10.1186/2051-4190-24-3

**Published:** 2014-02-10

**Authors:** Christophe Frainais, Caroline Kannengiesser, Martine Albert, Denise Molina-Gomes, Florence Boitrelle, Marc Bailly, Bernard Grandchamp, Jacqueline Selva, François Vialard

**Affiliations:** Laboratoire Clement, Le Blanc Mesnil, F-93110 France; Department of Genetics, Hôpital Bichat, Paris, F-75018 France; EA 2493, University of Versailles Saint-Quentin, Versailles, F-78035 France; Department of Reproductive Biology, Cytogenetics, Gynecology and Obstetrics, Poissy Saint Germain Hospital, Poissy, F-78303 France

**Keywords:** *RHOXF2*, Positive selection pressure, Infertility, Gene cluster, Homoedomain, *RHOXF2*, Pression de selection positive, Infertilité, Cluster de gènes, Homéodomaine

## Abstract

**Introduction:**

Genes involved in testicular differentiation, spermatogenesis, proliferation and apoptosis of germ cells have been shown to evolve rapidly and display rapid DNA changes. These genes are therefore good candidates for explaining impairments in spermatogenesis. Initial studies of some of these genes appear to confirm this hypothesis. The *RHOXF2* candidate gene belongs to the RHOX family clustered in Xq24 and is specifically expressed in the testis. It contains four exons and codes for a 288 amino acid (aa) transcription factor. It has a high degree of homology (>99.9%) with its paralogue *RHOXF2B*, which is also preferentially expressed in the testis.

**Objectives:**

To sequence *RHOXF2* and *RHOXF2B* in intracytoplasmic sperm injection (ICSI) patients and identify any single-nucleotide polymorphisms (SNPs) associated with impaired spermatogenesis.

**Materials:**

A cohort of 327 patients in ICSI programmes at Poissy and Bichat hospitals. All patients gave their written, informed consent to participation. One hundred patients had unaffected spermatogenesis and 227 displayed impaired spermatogenesis.

**Methods:**

The four exons in each of *RHOXF2* and *RHOXF2B* were sequenced in 47 patients with oligospermia or non-obstructive azoospermia. Given that exons 2 and 3 were found to harbour most of the SNPs, only these two exons were sequenced in the remaining 280 subjects.

**Results:**

Due to the extremely high degree of sequence identity between RHOXF2 and RHOXF2B, we were not able to distinguish between the sequences of these two genes. Although 9 SNPs were identified, there were no significant frequency differences between ICSI patients with normal vs. impaired spermatogenesis. Two insertions were identified: a 21-nucleotide insertion was retrieved in both groups and a guanine insertion (inducing a premature stop codon) only found in two patients with impaired spermatogenesis.

**Conclusion/outlook:**

*RHOXF2* is a good candidate for rapid evolution by positive selection. Analysis of the polymorphism frequency in exons 2 and 3 did not allow us to correlate the identified SNPs with male infertility. However, a single nucleotide insertion was identified only in men with impaired spermatogenesis. Further work will be needed to establish whether genetic changes in *RHOXF2* can give rise to defects in spermatogenesis.

**Electronic supplementary material:**

The online version of this article (doi:10.1186/2051-4190-24-3) contains supplementary material, which is available to authorized users.

## Résumé

**Introduction:**

Les gènes impliqués dans la différenciation des testicules, la spermatogenèse, la prolifération et l'apoptose des cellules germinales ont été montrés comme ayant une évolution rapide de la séquence d’ADN. Ces gènes sont donc de bons candidats pour expliquer les déficiences de la spermatogenèse. Les premières études semblent confirmer cette hypothèse. Le gène *RHOXF2*, appartenant à la famille des gènes RHOX avec un cluster dans Xq24, est un bon candidat car spécifiquement exprimé dans les testicules. Ce gène a un degré élevé d’homologie (> 99,9%), avec son paralogue *RHOXF2B* , qui est également exprimé préférentiellement dans les testicules.

**Objectifs:**

Séquencer *RHOXF2* chez des patients infertiles bénéficiant d’une injection intracytoplasmique de spermatozoïdes (ICSI) afin d’identifier des polymorphismes associés à une déficience de la spermatogenèse.

**Matériels:**

Une cohorte de 327 patients inclus dans un programme d’ICSI. Tous les patients ont donné leur consentement écrit et éclairé à la participation de cette étude. Cent patients n’avaient pas d’altération de la spermatogenèse et 227 avaient une déficience.

**Méthodes:**

Les quatre exons de *RHOXF2* ont été séquencés chez 47 patients présentant une oligospermie ou une azoospermie non obstructive. Étant donné que les exons 2 et 3 ont été trouvés comme ayant le plus de SNPs, seuls ces deux exons ont été séquencés dans les 280 sujets restants.

**Résultats:**

Bien que 9 SNPs aient été identifiés, il n’y avait pas de différence de fréquences significatives entre les patients ayant une altération, ou non de la spermatogenèse. Deux insertions ont été identifiées: une insertion de 21 nucléotides retrouvées dans les deux groupes et une insertion d’une guanine (induisant un codon stop prématuré) chez deux patients présentant une altération de la spermatogenèse.

**Conclusion:**

*RHOXF2* est un bon candidat pour une évolution rapide par sélection positive. L’analyse de la fréquence des polymorphismes dans les exons 2 et 3 ne nous permet pas actuellement de corréler les SNP identifiés avec l’infertilité masculine. Cependant, une insertion d’un seul nucléotide a été identifiée uniquement chez des hommes avec une déficience de la spermatogenèse. Des travaux complémentaires seront nécessaires pour déterminer l'impact du gène *RHOXF2* sur la spermatogenèse.

## Introduction

**Figure 1:**
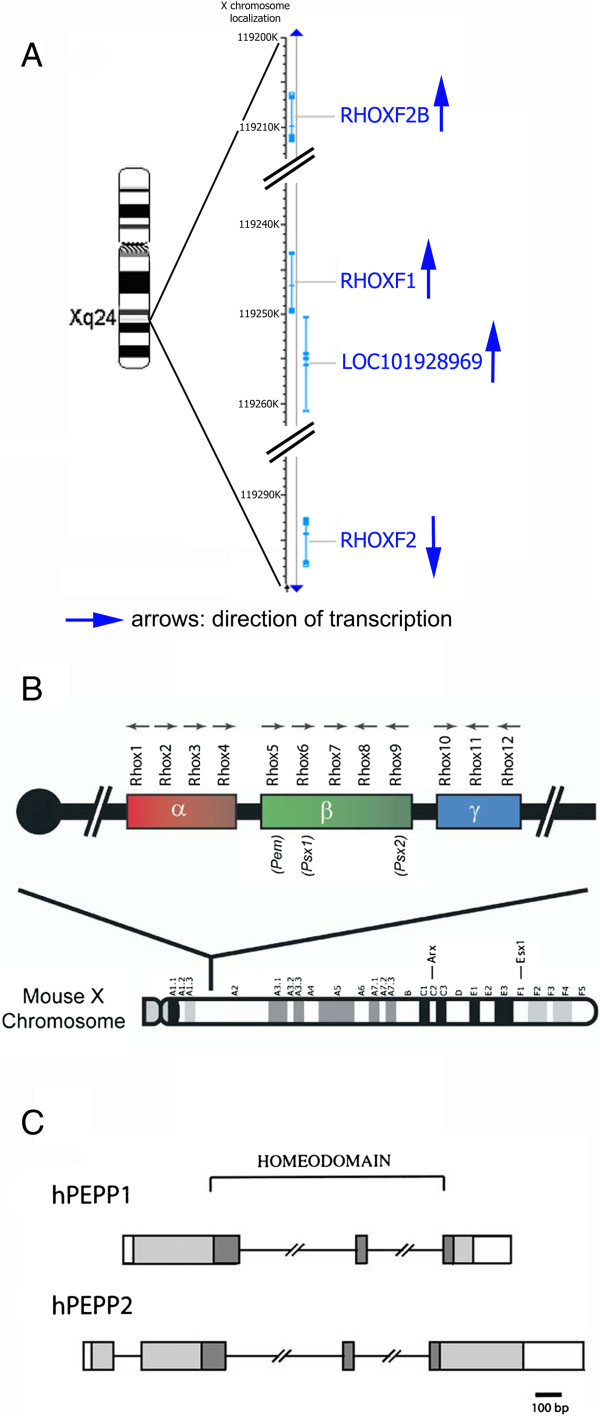
**RHOX genes. A**. Human RHOX cluster. **B**. Mouse RHOX cluster (from Mc Lean et al. [13]). **C**. Human RHOXF1 and RHOXF2 (from Wayne et al. [12]).

The study of genetic divergence between humans and chimpanzees [1] revealed the existence of certain groups of rapidly evolving genes subject to positive selection. This translates into a relatively high frequency of nucleotide sequence changes and then amino acid substitutions. More recently, studies of human single nucleotide polymorphisms (SNPs) have shown that some human genes have evolved in response to positive selection pressure [2]. This mechanism seems to preferentially affect genes involved in gametogenesis, apoptosis and immunity. Of these candidate genes, *RHOXF2*, *USP-26*, *Spo-11* and *PRM1* and *PRM2* are involved in different stages of spermatogenesis, *NROB1* and *REα* are involved in testicular differentiation, and *HYAL3* and *TSARG1* are involved in the proliferation/apoptosis of germ cells. Due to the presence of a large number of genes involved in the mechanisms of gametogenesis, it has been suggested that at least some of them have a role in infertility. Nielsen [1] hypothesized that mutations that increase cell proliferation and decrease germ cell apoptosis can sometimes be detrimental for the development of other parts of the body and may thus be subject to genomic conflict. This phenomenon might be partly responsible for the positive selection of these genes. Studies comparing infertile men with impaired spermatogenesis (oligospermia or azoospermic of secretory origin) with a control population of patients with normal spermatogenesis have already been undertaken. The preliminary results appear to confirm the involvement of genes such as *USP26*[3], *PRM1, PRM2*[4–6], *TNP1*, *TNP2*[7], *Spo11*[8, 9] and REα [10] in impaired spermatogenesis. Using a candidate gene approach, we chose to study the *RHOXF2* gene (also known as *PEPP-2* for *Pem, Esx1, Psx1, PSx2*). *RHOXF2* is a member of the RHOX family of genes located in Xq24. It features a the *Pem* DNA homeobox sequence encoding a 60 amino acid (aa) homeodomain protein that interacts with DNA. The protein is expressed in Sertoli cells [11, 12] and is involved in germ cell development in mice [11–13]. The RHOX gene family forms a cluster on the X chromosome in humans (*RHOXF1*, *RHOXF2* and *RHOXF2B*) (Figure 1) and in mice (comprising 12 genes). This family is characterized by two introns that are always located at the same point within the homeodomain. *RHOXF2* has four exons and encodes a transcription factor that is specifically expressed in the testis. Although the transcription factor’s role is not known in humans, the murine *Rhox 5/Pem* gene (an orthologue of *RHOXF2*) is involved in sperm maturation [13]. It encodes a 288 amino acids protein with a high glutamic acid N-terminal domain and a proline-rich domain in the C terminal [14]. When comparing the human and *Pan troglodytes* chimpanzee *RHOXF2* sequences, 21 differences have been described: 3 in exon 1, 8 in exon 2, 2 in exon 3 and 7 in exon 4. In human exon 2, there is also a 21 bp (CAG CAGAAC CCC GTC ACC GCC) insertion at nucleotide 322. In humans, the *RHOXF2* and *RHOXF2B* paralogues have a very high degree of sequence homology (>99.9%) but has the opposite orientation on the X chromosome. The currently known differences between the two genes are an adenine-thymine substitution at codon 93 and a thymine-cytosine substitution at codon 131 [14]. To date, few polymorphisms have been described for this gene [14]. More recently, [15], a study of *RHOXF2* copy number in primates showed that duplication only occurred in humans and four Old World monkey species (with at least 6 copies in chimpanzees). Furthermore, there was evidence of on-going selection for *RHOXF2*. Recently, it has been shown that the *RHOXF2/2B* genes are selectively expressed in the testis - primarily in germ cells in the adult testis but also in prespermatogonia in the human foetal testis. *RHOXF2/2B* mRNA expression increases in the second trimester during the development of the foetal testes (when gonocytes differentiate into prespermatogonia) [16]. The objective of the present study was to test for the involvement of *RHOXF2* genes in male infertility of secretory origin. To this end, we implemented a two-step strategy. In order to confirm the presence of new sequence variants, we first sequenced the *RHOXF2/2B* genes in a group of patients with impaired spermatogenesis. We then compared cases and controls in terms of the frequency of the newly identified SNPs.

## Material and methods

Population (Table 1): We sequenced RHOXF2 in 327 primary infertility patients participating in *in vitro* fertilization (IVF) programmes at Poissy and Bichat hospitals. The patients were divided into two groups as a function of the alteration in spermatogenesis (Table 1). Group 1 comprised patients with normal sperm characteristics (according to the WHO criteria [17]; sperm count >39 × 10^6^/ejaculate or >15 × 10^6^/ml) participating in an intracytoplasmic sperm injection (ICSI) program for conventional IVF failures (Group 1a, n = 73) and patients with obstructive azoospermia and normal spermatogenesis (Group 1b, n = 27). All group 1b patients had a normal testicular volume, urinary tract abnormalities (as confirmed by clinical and ultrasound examinations), alterations in at least one semen biochemistry parameter and normal testicular histology. Group 2 comprised patients with oligospermia (sperm count <39 × 10^6^/ejaculate or <15 x 10^6^/ml) (group 2a, n = 168) or non-obstructive azoospermia (Group 2b, n = 59). All azoospermic patients had altered testicular histology, normal semen markers, low testicular volume (volume < 10 ml) and no chronic pelvic inflammatory disease (i.e. a negative semen culture and fewer than 1 × 10^6^ round cells per ejaculate).

**Table 1:** **Patients’ testicular and hormonal parameters**

Groups	1: Normal spermatogenesis		2: Altered spermatogenesis	
***Settings***	a: Control	b: Obstructive azoospermia	a: Oligospermia	b: Non obstructive azoospermia
Patients number	73	27	167	60
Semen Volume (ml)	4.1* ± 1.7**	2.8 ± 2.2	3.6 ± 1.8	3.5 ± 1.4
Sperm count (M/ml)	68.8 ± 61.7	/	2.7 ± 3.6	/
Motility (a + b) (%)	54.2 ± 10.4	/	22.9 ± 16.4	/
Normal form (%)	43.2 ± 10.4	/	11.9 ± 11.3	/
FSH (UI/l)	5.0 ± 3.9	3.9 ± 1.9	11.9 ± 8.5	21.2 ± 13.5
LH (UI/l)	3.3 ± 1.4	3.2 ± 1.4	5.7 ± 3.1	7.5 ± 4.7
Total testosterone (ng/ml)	4.8 ± 2.3	4.8 ± 1.7	4.5 ± 1.8	4.0 ± 1.5
Inhibine B (pg/ml)	172.5 ± 69.0	158.4 ± 79.0	81.2 ± 60.1	33.3 ± 47.3

*mean; **standard deviation.

Infertile patients had undergone extensive assessment, including family and personal medical histories, physical and/or ultrasound examinations, karyotyping and Y deletion analysis. Testicular histology parameters and semen biochemical parameters (L-carnitine, fructose and citric acid) were analyzed in azoospermic patients. Sperm parameters were evaluated according to standard criteria (WHO, 1999). The hormone profile (FSH, LH, inhibin B and total testosterone) was determined in all groups of patients. All patients had a normal blood karyotype and no chromosome Y microdeletions. All participants gave their written, informed consent to participation in the study (which had been approved by an independent ethics committee CPP Ile de France XI) during pre-IVF genetic counselling.

The major exclusion criteria were as follows: age >50, vasectomy, endocrine disorders (hypogonadotropic hypogonadism), hormone therapy, toxic habits (>40 cigarettes per day, alcohol abuse and/or drug abuse), immunosuppressive treatment, chemotherapy and/or radiotherapy, unilateral orchidectomy, abnormal karyotype, Y chromosome deletions and chronic genital tract infections.

In order to identify potential polymorphisms and mutations, we initially sequenced all four *RHOXF2* exons in 47 patients with oligospermia or non-obstructive azoospermia (i.e. from groups 2a and 2b). We then sequenced exons 2 and 3 in the remaining 280 participants. All genetic variations were compared with the 1000 Genomes (http://browser.1000genomes.org/Homo_sapiens) and Exome Variant Server (EVS) (http://evs.gs.washington.edu/EVS/) databases.

Genomic DNA was extracted from blood samples using the Wizard® GenomicDNA Purification kit (Promega, Southampton, UK).

### Primer design

The primer pairs for each of the four exons were selected using Oligo software (Molecular Biology Insights, Inc. Cascade, CO, USA). The forward and reverse primer sequences consisted of 21 to 30 nucleotides and were selected 25–30 nucleotides upstream and downstream of the exons (with a minimum number of G/Cs, in order to prevent dimer formation) (Table 2).

**Table 2:** **Primers sequences**

Exon	Primer	
1	Forward	AGACACCAGAAGAACGTTGCAGGC
	Reverse	TGAGAGCAGTGGTAGTGCAGGGGT
2	Forward	CATAGCCCCACTACAGAACCCACTAGG
	Reverse	ACTGGATGATACCCCTGCACTACCACT
3	Forward	TGAGGCTCAGCTGGGTGTTACAGAAC
	Reverse	TTGCCTATACATGAAAAAGGCCACAA
4	Forward	CACTAGGGGAAAGGTGTGGTGGTCA
	Reverse	TACAAGTGCCCTAAAAATGGGTCAATGAT

### DNA amplification

PCRs were performed on 1 μl of native DNA with Taq Gold ®, 3 μl 25 mM MgCl2, and 1 μl 5 mM dNTP. A total of 35 cycles were performed (denaturation at 96°C for 30 s; hybridisation at 60°C for 30 s (exons 1 and 2) or 57°C for 30 s (exons 3 and 4); elongation at 72°C for 60 s). The PCR fragments were then purified enzymatically with ExoSAP (USB, Cleveland, Ohio, USA).

### DNA sequencing

DNA was sequenced using 1 μl Big Dye Terminator on an Eppendorf thermocycler with the following program: hot start: 96°C for 5 min; 25 cycles (denaturation at 96°C for 30 s, hybridization at 50°C for 15 s, elongation at 60°C for 4 min); final elongation: 60°C, 10 min. After purification on a Sephadex G50 plate Millipore column, raw sequences were analyzed and interpreted with Seqscape® software (Applied Biosystems, Life Biotechnologies, NY, USA).

### Statistical analysis

Intergroup comparisons of polymorphism and haplotype frequencies were performed with Fisher’s exact test and the chi-squared test. The threshold for statistical significance was set to p < 0.05. All analyses were performed with Statview software (SAS Institute Inc., Cary, NC, USA).

## Results (Table 3)

**Table 3:** **Exons 2 and 3 SNPs frequencies**

SNP and DNA change		Previously described	Variant type genetic	Group 1	Group 2	p(I vs II)=
(Included in the homeodomain)			Variation at protein level	(n = 100)	(n = 227)	
				n (%)	n (%)	
c.202G > A		rs148604152	Missense p.G68R	1(1.0)	1(0.4)	0.5188
c.225_245dup,		No	p.Glu76_Gly82dup	1 (1.0)	3 (1.3)	1
c.267A > G		rs149340601	Synonymous p.L89=	2 (2.0)	0	0.0929
c.277G > A	all	rs146311958	Missense p.D93N	75 (75.0)	176 (77.5)	0.6703
	Homozygote			1 (1.0)	2 (0.9)	1
c.381dupG		Yes no rs	Frameshift p.L128Afs*34	0	2 (0.9)	0.8617
c.381C > T		No	Synonymous p.G127=	0	1 (0.4)	1
c.396C > T		rs199940228	Synonymous p.A133=	0	1 (0.4)	1
**c.411C > T**		rs142963365	Synonymous p.N137=	0	1 (0.4)	1
**c.451C > T**		no	Missense p.R151C	0	2 (0.9)	1
**c.452G > A**	**all**	rs142899626	Missense p.R151H	40 (40.0)	98 (43.2)	0.6281
	**Homozygote**			0	1 (0.4)	1
**c.526C > T**		rs199871532	Missense p.L176F	10 (10.0)	17 (7.5)	0.5136

First, all four *RHOXF2* exons were sequenced in 47 patients with oligospermia or non-obstructive azoospermia. The sequences corresponded to *RHOXF2*. In view of the high degree of sequence homology (>99.9%), a pseudo-biallelic nomenclature was adopted for the SNPs’ genotype. Five previously described heterozygous SNPs [14] were identified: 3 on exon 2, one on exon 3 and one on exon 4. These SNPs were also present in the 1000 Genomes and EVS databases. A previously described frame-shift variant in exon 2 (a guanine insertion leading to a premature stop codon (TGA) of the cDNA (c.381dupG).p.(L128Afs*34)) was also identified. Given that we did not find any new SNPs in exons 1 and 4, we decided to only study exons 2 and 3 in the remaining 280 patients. The overall study population therefore comprised 100 patients from group I and 227 patients from group II. We identified five heterozygous SNPs (all located in exon 2) in our population. Three had been reported previously and two had not. One of the latter was a 21-nucleotide duplication (G**AG AAG AAA AAG ATG GCG GCGG**CC) from nucleotide 246 onwards; this leads to a 7 aa protein duplication (QKKKMAA) at aa 82 and thus a 295 aa protein). This duplication (official nomenclature: c.225_245dup,p.(Glu76_Gly82dup)) was found in one patient from group I and three patients from group II. A c.381dupG was found in one patients with impaired spermatogenesis. Overall, four SNPs were synonymous and five were non-synonymous. Furthermore, two insertions (one of which led to a premature stop codon) were identified only in patients with spermatogenesis failure. There were no intergroup differences in SNP frequency (Table 3) and no haplotype differences were observed when combining the three most frequent SNPs (p.D93N, R151H and p.L176F). Three patients were homozygous for D93N and one was homozygous for R151H.

Sperm characteristics were for the two oligospermic patients with the c.381dupG as follow : Patient 1: sperm count: 2.20 x10^6^/ml, motility: 38%, typical form: 0%; Patient 2: sperm count: 4/ml, .motility and typical form not performed.

Considering all subgroups separately (Additional file 1: Table S1), the only significant frequency difference was observed for p.D93N (the most frequent SNP, found in 74.2% of the study population) comparing azoospermic (73.8%) and oligospermic patients (88.1%) with spermatogenesis failure (p = 0.0288).

## Discussion

Genetic divergence between Homo sapiens and the chimpanzee may result from positive selection pressure (notably on genes involved in immunity, gametogenesis and apoptosis) [1, 2]. The positive selection pressure on male gametogenesis is higher in humans than in the chimpanzee [18]. Rapid evolution of these genes leads to changes in one or more nucleotides, which in turn can impair the function of the corresponding protein and, in some cases, cause infertility. In humans, the X and Y chromosomes are particularly exposed to this selection pressure because of the absence of Y homologous recombination during meiosis and low recombination rates (50%) for the X chromosome. It has also been shown that male infertility can be related to Y chromosome defects [19–23]. One can therefore hypothesize that male infertility can be caused by polymorphisms in X chromosome genes involved in spermatogenesis. Mutations in the USP26 gene in Xq28 (also considered to be subject to positive selection pressure) were found in 7% of a series of patients with Sertoli cell-only syndrome [24]. The fact that other X chromosome polymorphisms (both SNPs [25] and copy number variations [26]) have been recently reported strengthens the hypothesis whereby they have an impact on spermatogenesis. The RHOX family gene is located in Xq24 and codes for transcription factors that are only expressed in the testes. Mice have 12 RHOX orthologues, which are also organised in a cluster [12, 13]. Some of these genes are only partially duplicated [27, 28]. Of these, Rhox5 is expressed in Sertoli cells and appears to have an important role in spermatogenesis. Impaired expression leads to azoo-teratospermia and increased apoptosis of germ cells. Humans RHOXF2 presents a high degree of sequence homology (63.8%) with the mouse Rhox5 gene [12]. Few *RHOXF2* polymorphisms have been identified and there are no data on their frequency. In humans, the *RHOXF2* and *RHOXF2B* paralogues have a very high degree of sequence homology (>99.9%). *RHOXF2B* is located less than 100 kb away from *RHOXF2* but has the opposite orientation. The human gene duplication is thought to have occurred very recently [14]. Due to the very high degree of homology (99.9%), there are no data on the differential expression of these two paralogues. A recent study of sequence and copy number variations of *RHOXF2* in humans and a non-human primates showed a parallel gene duplications/losses in multiple primate lineages [15]. Eleven non-human primate species have only one *RHOXF2* copy, two copies are present in humans and four Old World species of monkey and at least six copies are present in chimpanzees. The gene duplication in primates was probably mediated by non-allelic recombination via flanking endogenous retroviral sequences [15]. Furthermore, an analysis of eight non-synonymous variant sites in humans has suggested that the gene selection process is on-going. Furthermore, it was recently reported that *RHOXF2B* is selectively expressed in male germ cells [16]. These various datasets support the hypothesis whereby *RHOXF2* variants may be present in patients with impaired spermatogenesis. Our two-step strategy (based on the preliminary identification of new variants in a small group of patients with impaired spermatogenesis and then extension of the analysis to control patients) enabled us to identify (i) new synonymous and missense variants and (ii) two frame-shift variants (one of which was associated with a premature stop codon). However, patients with and without impaired spermatogenesis did not differ in terms of the frequency of these polymorphisms. We established haplotypes on the basis of the three most frequent polymorphisms but again did not observe any differences in frequency between the two groups of patients. These findings suggest that there is no correlation between the frequency of *RHOXF2* polymorphisms and impaired spermatogenesis. However, this result is preliminary because our study only covered RHOXF2′s coding sequences. It would be interesting to study the promoter regions in patients with impaired spermatogenesis. The *RHOXF2* promoter has a high degree of homology with that of the mouse Rhox5 gene [12], which contains an androgen binding site. The presence of polymorphisms in this region could therefore modulate *RHOXF2* transcription and reduce translation. In the mouse, low Rhox5 expression was shown to have a harmful effect on spermatogenesis [13]. Furthermore, we did not study exon 4, which carries a polymorphism that is defined as harmful by the PANTHER classification system (http://www.pantherdb.org). We identified two rare duplications in our patients with impaired spermatogenesis. The first duplication is a guanine insertion that shifts the reading frame, induces a premature stop codon and thus produces a truncated protein. This duplication was found in two patients with spermatogenesis failure and none of the control patients. The EVS database lists this duplication for only one individual, the fertility of which is not stated. Given that Rhox gene mutation alter testicular function in rodents [27], this variant may indeed be pathogenic. The second duplication is located in exon 2. This is a duplication of a 21-bp fragment from base 165, resulting in the insertion of a 7 aa polypeptide (but no change in the homeodomain). This mutation was identified in three patients with impaired spermatogenesis and in one control patient.

Except for the frameshift mutation p.L128Afs*34 which disrupts the homeodomain (from amino acid 134 to 193), the others SNPs do not exhibit a specific phenotype. This may be due to several aspects. Firstly, seven of the 11 reported SNPs were outside the homoeodomain. Secondly, one of the remaining four SNPs is synonymous. For 2 of the others, the substituted amino acid is quite similar to the native amino acid and thus does not induce a great change in homeodomain’s conformation (the basic amino acid arginine and histidine for p.R151H and the non-polar leucine and phenylalanine amino acids for p.L176F). The most interesting variation is probably p.R151C, in which arginine is replaced by the dissimilar cysteine. A change in the homeodomain’s interaction with DNA cannot therefore be ruled out. When compared with the two previously mentioned SNPs, p.R151C was only observed in the group of patients with spermatogenesis failure. However, the very low frequency explains the absence of a statistically significant difference (as was is also the case for the frameshift mutation p.L128Afs*34). Thirdly, the amino acid positions 151 and 176 were not conserved across orthologues [12]. Hence, these various points may explain why negative results were observed in our series and, in particular, why the RHOXF2 SNPs did not appear to predispose their bearers to infertility.Due to technical limitations and the very high degree of homology between *RHOXF2* and *RHOXF2B*, we could not distinguish between *RHOXF2* or *RHOXF2B* polymorphisms. It is not yet known whether the two paralogues have distinct functions or complement each other perfectly. It is therefore impossible to determine the relative impact of the different polymorphisms or duplications on spermatogenesis. In order to confirm our present results, it would be interesting to sequence exon 2 in a larger cohort of patients and controls. An analysis of *RHOXF2* and *RHOXF2B* expression patterns would also be very interesting but would require us to be distinguish between the two paralogues.

## Conclusion

Mutations in the *RHOXF2* and *RHOXF2B* genes (which encode homeodomain proteins exclusively expressed in the testis) might explain some cases of male infertility. Our study confirmed the presence of the polymorphisms identified by Wayne [14] and a number of new polymorphisms. A truncating mutation (caused by insertion of a nucleotide) was encountered only in patients with impaired spermatogenesis. Although several polymorphisms were identified in *RHOXF2* and *RHOXF2B* (suggesting recent positive selection pressure), there were no significant difference between patients with impaired spermatogenesis and control patients in terms of the polymorphism frequencies. It would nevertheless be interesting to (i) continue this analysis of *RHOXF2* and *RHOXF2B* in a larger population, (ii) sequence exon 4 and the promoter sequence and (ii) study other testis-active genes located on chromosome X, (such as *PEPP-1* (*OTEX*) [14, 29], *ESX1-R*[30], *ESX-1 L*[31, 32], *TGIFL-X*[33]) or genes that have been subjected to positive selection pressure. If possible, specific analysis of *RHOXF2* and *RHOXF2B* expression should tell us whether both genes are expressed exclusively in the testis and which of the two paralogues is required for spermatogenesis.

## Electronic supplementary material

Additional file 1: Table S1: Exons 2 and 3 SNPs frequencies in all subgroups. (DOC 48 KB)

## Abbreviations

aa: Amino acid
SNPs: Single-nucleotide polymorphisms
ICSI: Intracytoplasmic sperm injection
IVF: *In vitro* fertilization.
