# Supplementary material for: RHOXF2 gene, a new candidate gene for spermatogenesis failure
Source: Basic Clin Androl. 2014 Feb 10;24:3. doi: 10.1186/2051-4190-24-3 (PMC4349744; doi:10.1186/2051-4190-24-3)
Supplement: Supplementary file 1 — Additional file 1: Table S1: Exons 2 and 3 SNPs frequencies in all subgroups. (DOC 48 KB) [file 12610_2013_17_MOESM1_ESM.doc]

**Additional file 1: Table S1. Exons 2 and 3 SNPs frequencies in all subgroups:**

| SNP and DNA change  **(Included in the homeodomain)** | | Previously described | Variant type genetic  Variation at protein level | Group Ia  (n=73) | Group Ib  (n=27) | Group IIc  (n=167) | Group IId  (n=60) | Statistical significance |
| --- | --- | --- | --- | --- | --- | --- | --- | --- |
|  |  |  |  | n (%) | n (%) | n (%) | n (%) |  |
| c.202G>A | | [rs148604152](http://www.ncbi.nlm.nih.gov/projects/SNP/snp_ref.cgi?searchType=adhoc_search&type=rs&rs=148604152) | Missense p.G68R | 1 (1.4) | 0 | 1 (0.6) | 0 | No |
| c.225_245dup, | | No | p.Glu76_Gly82dup | 1 (1.4) | 0 | 3 (1.8) | 0 | No |
| c.267A>G | | [rs149340601](http://www.ncbi.nlm.nih.gov/projects/SNP/snp_ref.cgi?searchType=adhoc_search&type=rs&rs=149340601) | Synonymous p.L89= | 1 (1.4) | 1 (3.7) | 0 | 0 | No |
| c.277G>A | All | [rs146311958](http://browser.1000genomes.org/Homo_sapiens/Variation/Mappings?db=core;g=ENSG00000131721;r=X:119292467-119297945;t=ENST00000371388;v=rs146311958;vf=37243373;source=dbSNP) | Missense p.D93N | 52 (71.2) | 23 (85.2) | 124 (74.2) | 52 (86.7) | (1) |
|  | Homozygote |  |  | 1 (1.4) | 0 | 1 (0.6) | 1 (1.7) | No |
| c.381dupG | | Yes no rs | Frameshift p.L128Afs*34 | 0 | 0 | 2 (1.2) | 0 | No |
| c.381C>T | | No | Synonymous p.G127= | 0 | 0 | 1 (0.6) | 0 | No |
| c.396C>T | | [rs199940228](http://browser.1000genomes.org/Homo_sapiens/Variation/Mappings?db=core;g=ENSG00000131721;r=X:119292467-119297945;t=ENST00000371388;v=rs199940228;vf=54610748;source=dbSNP) | Synonymous p.A133= | 0 | 0 | 1 (0.6) | 0 | No |
| **c.411C>T** | | [rs142963365](http://www.ncbi.nlm.nih.gov/projects/SNP/snp_ref.cgi?searchType=adhoc_search&type=rs&rs=142963365) | Synonymous p.N137= | 0 | 0 | 1 (0.6) | 0 | No |
| **c.451C>T** | | [no](http://browser.1000genomes.org/Homo_sapiens/Variation/Mappings?db=core;g=ENSG00000131721;r=X:119292467-119297945;t=ENST00000371388;v=TMP_ESP_X_119293304;vf=57077449;source=ESP) | Missense p.R151C | 0 | 0 | 2 (1.2) | 0 | No |
| **c.452G>A** | **All** | [rs142899626](http://www.ncbi.nlm.nih.gov/projects/SNP/snp_ref.cgi?searchType=adhoc_search&type=rs&rs=142899626) | Missense p.R151H | 26 (35.6) | 14 (51.9) | 71 (42.5) | 27 (45.0) | No |
|  | **Homozygote** |  |  | 0 | 0 | 1 (0.6) | 0 | No |
| **c.526C>T** | | [rs199871532](http://browser.1000genomes.org/Homo_sapiens/Variation/Mappings?db=core;g=ENSG00000131721;r=X:119292467-119297945;t=ENST00000371388;v=rs199871532;vf=54544694;source=dbSNP) | Missense p.L176F | 9 (12.3) | 1 (3.7) | 9 (5.4) | 8 (13.3) | No |

(1) IIc vs IId : p=0.0481 and Ia+IIc vs Ib + IId : p=0.0149
